# Effects of Multi-Role Collaborative Palliative Care on Anxiety, Cancer-Related Fatigue, and Quality of Life in Patients With Advanced Lung Cancer

**DOI:** 10.62641/aep.v54i2.2174

**Published:** 2026-04-15

**Authors:** Ying Hou, Hui Xu, Yunsa Huo, Hequn Li, Yue Tian, Qin Wei

**Affiliations:** ^1^Department of Medical Oncology, Nanjing Lishui People's Hospital, 211200 Nanjing, Jiangsu, China

**Keywords:** cancer-related fatigue, anxiety, multidisciplinary care, palliative care, lung cancer

## Abstract

**Background:**

This research aimed to examine the effects of multi-role collaborative palliative care on anxiety, cancer-related fatigue (CRF), psychological status, and quality of life in patients with advanced lung cancer, and to explore potential mechanisms using correlation analysis and structural equation modelling.

**Methods:**

We conducted a retrospective study of the medical records of 200 patients with advanced lung cancer. Based on the care they received, patients were divided into two groups: a control group (n = 100) that received standard nursing care, and a combined nursing group (n = 100) that received multi-role collaborative palliative care. Anxiety, CRF, psychological status, and overall quality of life were compared between the two groups based on the record documented before and after nursing care. Adverse events recorded during the nursing period were also reviewed and analysed. Path analysis of variables was conducted using the AMOS module of SPSS.

**Results:**

Following nursing care, the combined group showed significantly lower CRF and anxiety scores, and significantly higher psychological state and quality of life scores compared with both pre-nursing scores and the control group (all *p* < 0.05). There was no statistically significant difference in the rate of adverse event between the two groups (36.00% vs. 28.00%, *p* > 0.05). Path analysis indicates that multi-role collaborative palliative care is associated with lower levels of anxiety. This association has a direct relationship and indirect relationships through its connection with the reduction of CRF and the improvement of psychological condition. Path analysis shows that multi-role collaborative palliative care not only directly alleviates patients' inner anxiety, but also may indirectly reduce inner anxiety by lowering CRF and improving psychological conditions.

**Conclusions:**

Implementing multi-role collaborative palliative care for patients with advanced lung cancer can help alleviate CRF, relieve anxiety, improve psychological state and enhance quality of life. Exploratory path analysis suggests that this nursing model has a significant direct statistical association with lower anxiety. This association may also involve indirect interrelations with lower CRF and a better psychological state.

## Introduction

Lung cancer ranks as the second most common form of cancer globally [1], and its incidence continues to increase annually [2]. Because the illness rarely shows clear warning signs at first, the disease often progresses insidiously, resulting in most patients being diagnosed at an advanced stage, which substantially compromises treatment efficacy [3, 4]. In recent years, advances in antitumour therapies have modestly prolonged the survival of patients with advanced lung cancer [5]; the 3-year survival rate of lung cancer patients increased from 19% in 2001 to 31% during 2015–2017, while the median survival extended from 8 to 13 months [6]. Nevertheless, the overall prognosis remains unfavourable. Cancer-related fatigue (CRF) is one of the most prevalent and distressing symptoms in this population, occurring in 39%–90% of patients undergoing anticancer treatment [7]. Evidence indicates that, compared with other tumour types, lung cancer patients experience a heavier burden of fatigue, with 81.5% of advanced lung cancer cases experiencing varying degrees of fatigue [8]. The persistent CRF not only impairs the physical functions of patient, but also leads to anxiety, thereby reducing their quality of life. At the same time, tumour treatment is often accompanied by obvious toxic side effects. Coupled with the rapid progression and uncertain prognosis of advanced lung cancer, patients are more likely to experience anxiety [9, 10]. Severe anxiety further increases the psychological burden of patient, affecting their treatment compliance and prognosis [11]. Therefore, it is of critical importance to implement appropriate care for patients with advanced lung cancer, aiming to alleviate anxiety and CRF, improve quality of life, and ensure optimal treatment outcomes.

Palliative care, a specialised healthcare approach for individuals with terminal or life-limiting conditions, emphasises multidisciplinary collaboration and addresses physical, psychological, social, and spiritual needs, with the goal of alleviating symptoms and reducing suffering [12]. Previous study has demonstrated that, compared with conventional oncological care, early integration of palliative care nursing can substantially improve emotional well-being and quality of life in patient with advanced lung cancer, and may even extend survival to some extent [13]. Multi-role collaborative care, a patient-centred approach, highlights the involvement of a multidisciplinary team consisting of physicians, nurses, psychologists, and other healthcare professionals [14]. Research suggests that collaborative nursing involving multiple roles and disciplines can provide synergistic benefits in symptom management, psychological support, and social care, making it particularly suitable for patients with complex conditions and diverse needs [15]. However, evidence regarding the application of multi-role collaborative palliative care in patients with advanced lung cancer remains limited. Accordingly, the present study was undertaken to explore the effects of this care model on anxiety, CRF, and quality of life in patients with advanced lung cancer, and to further delineate its potential interrelations among these variables, thereby providing valuable insights for optimising clinical nursing practices.

## Materials and Methods

### Study Population

This study was a retrospective consecutive enrolment study. A total of 200 patients with advanced lung cancer who were treated at Nanjing Lishui People’s Hospital from December 2021 to December 2024 and met the inclusion and exclusion criteria were included. Based on the care approach administered, the cohort was divided into two groups: a control group (n = 100) receiving standard nursing care and a combined nursing group (n = 100) undergoing multi-role collaborative palliative care.

The inclusion criteria were as follows:(1) diagnosis of stage III or IV lung cancer according to the Chinese Medical Association Guidelines for Clinical Diagnosis and Treatment of Lung Cancer (2019 edition) [16], with histopathological confirmation of malignancy; (2) age ≥18 years; (3) presence of measurable solid tumour lesions on imaging; (4) no recent use of psychotropic medications; (5) fulfilment of indications for palliative care; (6) availability of complete clinical data; and (7) no impairment of speech or hearing, and ability to communicate normally.

The exclusion criteria were as follows: (1) patients unwilling to acknowledge their disease condition or with poor compliance; (2) patients with severe pain attributable to comorbid conditions; (3) patients with severe multi-organ failure and life expectancy <3 months; (4) presence of psychiatric disorders or consciousness disturbances; (5) withdrawal from the study midway; (6) complete bedridden status or requiring end-stage lung cancer care; and (7) concomitant malignancies or metastases to organs such as the brain or kidney.

### Nursing Protocol

Control group: Patients received routine nursing care for advanced lung cancer, including disease observation, monitoring of vital signs, basic daily care, medication guidance, and health education.

Combined nursing group: Patients received standard care identical to that of the control group, supplemented with multi-role collaborative palliative care:

(1) Establishment of a care team. The team consisted of a head nurse, nurses, attending physicians, psychologists, and rehabilitation therapists. The head nurse served as team leader, responsible for training members on concepts and practices related to palliative care. Only those who passed the post-training assessment were qualified to implement the nursing measures.

(2) Development of a care plan. Based on the baseline information recorded in patients’ medical records (including physical condition, psychological status, and quality of life), nurses jointly discussed and formulated individualized care plans, specifying objectives, priorities, and task allocation.

(3) Implementation of multi-role collaborative palliative care. The team cooperated according to the individualized plan, held regular multidisciplinary consultations, and continuously reassessed patients’ conditions and needs to adjust strategies in a timely manner. Nursing staff were responsible for daily symptom monitoring, basic care, and psychological support; psychologists provided counselling, relaxation training, or cognitive-behavioural therapy as appropriate; dietitians assessed nutritional status and designed dietary plans; and rehabilitation therapists guided mild exercise and pulmonary function training. Continuous communication with patients and families was maintained to encourage active participation in care decisions, with weekly records of nursing care and outcomes. The care team held at least one collective discussion per week to review overall patient status, ensuring continuity, individualization, and humane care.

(4) Quality control of nursing effectiveness: To ensure the standardised implementation of multi-role collaborative palliative care nursing, quality control measures were taken for the relevant nursing staff. Specialised quality control personnel conducted daily inspections of the nursing work, with the inspection times set at 10:00 and 16:00 every day. The inspection results were recorded in detail. The team leader conducted irregular inspections of the implementation of the nursing work every day. Existing problems and deficiencies were promptly corrected and handled, and the nursing measures were improved and optimised to enhance the quality of nursing. Specific measures are presented in Table 1. The nursing duration for both groups was one month.

**Table 1.** **Multi-role collaborative palliative care measures**.

Nursing measures	Specific measures	Responsible personnel
Psychological support	Disclosure and emotional support: With family consent, patients were informed of their condition in a gentle manner. The patient’s emotional state was evaluated once a day, each session lasting approximately 5 minutes. Through observing facial expressions, verbal communication, and behavioural responses, the emotional status was recorded. If anxiety or unease was detected, verbal comfort or emotional counselling was provided immediately until the patient’s emotional state stabilizes. At the same time, psychological counselling is provided to patients 1–2 times a day, each session lasting 15–20 minutes. This helped patients adopt a positive attitude towards the disease and treatment, encouraged them to express their true feelings, and assisted them in alleviating their anxiety at the end of life and reducing psychological unease	Nurses, psychological counsellors
	Peer support and interest diversion: Weekly patient support group meetings were held to share treatment experiences, each session lasting 30–40 minutes. Patients’ interests were engaged through music or television sessions 1–2 times per day, 10–20 minutes per session	Nurses
Pain management	Pain assessment and pharmacological treatment: Pain was assessed every 4 hours [using the Numerical Rating Scale (NRS)], and analgesics were administered according to physicians’ orders. For radiotherapy-induced skin pain, patients were advised to wear loose clothing and avoid friction; if redness or swelling occurred, medication was administered according to physicians’ instructions	Nurses, physicians, pharmacists
	Self-management of pain: Patients were taught self-assessment of pain, as well as self-suggestion or distraction techniques, twice daily, for 10–15 minutes each session	Nurses
Respiratory guidance and management	Relief of dyspnoea and airway maintenance: Respiratory rate, rhythm, and oxygen saturation were monitored each shift (every 8 hours), and trends were recorded. For each patient, the degree of breathing difficulty was evaluated using the numerical scoring method. If the score was ≥ 4, the attending doctor was notified immediately and appropriate treatment measures were initiated. Based on the blood oxygen saturation and clinical symptoms, oxygen therapy was given according to the doctor’s advice when the blood oxygen saturation was < 90% or obvious breathing difficulties occurred. The oxygen flow rate was adjusted to maintain a blood oxygen saturation of ≥ 92%, and a humidification device was used. During the nursing process, the effect of oxygen therapy was dynamically evaluated. When the blood oxygen saturation was stable and the breathing difficulty symptoms had significantly improved, the oxygen therapy was gradually reduced until it was stopped. At the same time, if the patient can tolerate and had no contraindications, the patient was assisted to adjust the body position to a semi-sitting position, forward-leaning sitting position, to reduce the degree of breathing difficulty, maintaining for 10–30 minutes until the breathing difficulty was relieved or the patient experienced discomfort. For patients who had difficulty in expectorating or whose sputum was thick, they were assisted them in performing active expectoration (such as chest tapping and aerosol inhalation) or suctioning when necessary. This was done 1–2 times a day, each session lasting 10–20 minutes, to facilitate the expulsion of sputum; if necessary, the frequency can be increased according to the condition of the sputum. For pleural effusion, thoracentesis or drainage was performed under physician guidance, with postoperative care of drainage tubes. Patients were instructed to perform diaphragmatic and pursed-lip breathing 2–3 times daily, 5–10 minutes per session, to improve respiratory efficiency	Nurses, physicians, rehabilitation therapists
Nutritional management	Nutritional support and dietary management: Patients’ body weight, oral intake, and serum albumin/prealbumin levels were monitored daily. For patients who had breathing difficulties or coughing that affected their eating, they were instructed to have smaller meals more frequently, with 3–5 meals per day, and to extend the eating time by 5–10 minutes for each meal. Patients with insufficient oral intake received nasogastric feeding or parenteral nutrition as prescribed by physicians, with each session lasting 30–60 minutes, administered 1–3 times per day, along with appropriate tube care. If tube feeding was refused, family members were guided to provide high-protein, high-calorie, easily digestible liquid or semi-liquid meals, approximately 200–300 mL per meal, 3–5 times daily. Dietary adjustments were made for each meal to address nausea or taste changes caused by chemo/radiotherapy, such as providing mildly sweet or low-Odor foods to increase appetite. The nursing staff assessed the effectiveness of nutritional management (once a week) and adjusted the nutritional plan in a timely manner based on the assessment results	Nurses, nutritionists

### Outcome Measures

(1) CRF: Data were extracted from patients’ medical records for CRF scores one day before and one month after nursing, which were assessed using the Chinese version of the Cancer Fatigue Scale (CFS) [17]. The CFS consists of 15 items across three dimensions (affective, physical, and cognitive fatigue), each scored on a 0–4 scale, with a total score of 60. Lower scores indicate milder fatigue. The Cronbach’s α coefficients for internal consistency of each dimension in the original scale and the total scale ranged from 0.63–0.86, and the Kaiser–Meyer–Olkin (KMO) value of the structural validity was 0.85, indicating good reliability and validity.

(2) Psychological status: Data were extracted from patients’ medical records for psychological status scores at the same time points as the CRF assessments, which were assessed using the Connor-Davidson Resilience Scale (CD-RISC), developed by Connor and Davidson [18]. This scale contains 25 items across three dimensions (optimism, strength, and resilience), scored on a scale of 0–4, with a total score of 100. Higher scores reflect better psychological resilience. The Cronbach’s α coefficient of the original scale was 0.91, indicating a high level of reliability.

(3) Anxiety: Data were extracted from patients’ medical records for anxiety scores at the same time points as the CRF assessments, which were assessed using the Self-Rating Anxiety Scale (SAS) developed by Zung [19]. The SAS consists of 20 items rated on a four-point Likert scale, with each item scored from 1 to 4, including five reverse-scored items. The sum of the 20 items yields the raw score, which is then multiplied by 1.25 and rounded to the nearest integer to obtain the standard score. Higher scores indicate more severe anxiety. Previous studies have shown that the original scale has good internal consistency reliability, with Cronbach’s α coefficients ranging from 0.70–0.85, and also has good structural validity.

(4) Quality of life: Data were extracted from patients’ medical records for quality-of-life scores at the same time points as the CRF assessments, which were assessed using the Chinese version of the Functional Assessment of Cancer Therapy-General (FACT-G) [20]. This instrument includes 27 items scored on a scale of 0–4, with a total score of 108. Higher scores indicate better quality of life. The Cronbach’s α coefficients of each domain in the original scale were all above 0.8, indicating high reliability. It is widely applicable for assessing the quality of life of patients with malignant tumours in China.

(5) Adverse reactions: Adverse events (including fatigue, somnolence, and pain) recorded during nursing were extracted from patients’ medical records, and their incidence was calculated.

(6) Through correlation analysis and structural equation modelling, we further explored the potential statistical associations and interrelations among the measured variables using the AMOS module of SPSS.

### Statistical Analysis

All statistical analyses were conducted using SPSS version 26.0 (IBM Corp., Armonk, NY, USA). Normality of continuous variables was evaluated via the Kolmogorov–Smirnov test. Variables with a *p* value > 0.05 were deemed normally distributed and reported as mean ± standard deviation (SD); the differences between the two groups before and after nursing were compared using independent sample t-tests, while the differences within the same group before and after nursing were analysed using paired t-tests. For variables with a *p* value < 0.05 (non-normally distributed), results were presented as median and interquartile range [M (P_25_, P_75_)], and intergroup comparisons were conducted using the Mann–Whitney U test. Categorical variables were expressed as frequencies and percentages [n (%)], with group comparisons performed via the chi-square (χ^2^) test. Associations between variables were analysed using Spearman’s rank correlation coefficient, and path analysis was executed with the Amos module of SPSS. Statistical significance was defined as a two-sided *p* value < 0.05.

## Results

### Comparison of General Clinical Characteristics Between Groups

There were no differences in baseline demographic and clinical features between the groups (*p* > 0.05, Table 2).

**Table 2.** **Baseline demographic and clinical characteristics of the two groups**.

Characteristics		Control group (n = 100)	Combined nursing group (n = 100)	Z/t/χ^2^	*p*
Age		67.09 ± 3.46	66.46 ± 3.44	1.293	0.198
Gender					
	Male	61 (61.00)	69 (69.00)	1.407	0.236
	Female	39 (39.00)	31 (31.00)		
BMI (kg/m^2^)		21.34 ± 2.44	21.67 ± 2.66	–0.915	0.361
TNM Stage					
	Stage III	39 (39.00)	45 (45.00)	0.739	0.390
	Stage IV	61 (61.00)	55 (55.00)		
Pathological type					
	Small-cell lung cancer	37 (37.00)	32 (32.00)	0.553	0.457
	Non-small cell lung cancer	63 (63.00)	68 (68.00)		
Disease duration (years)		5.00 (4.00, 5.00)	5.00 (4.00, 5.00)	–0.716	0.474
Education level					
	Junior high school or below	77 (77.00)	75 (75.00)	0.110	0.741
	Senior high school or above	23 (23.00)	25 (25.00)		
Marital status					
	Single	2 (2.00)	5 (5.00)	1.462	0.481
	Married / Cohabiting	71 (71.00)	71 (71.00)		
	divorced / Widowed	27 (27.00)	24 (24.00)		

Note: Categorical variables are expressed as n (%); Continuous variables are presented as mean ± SD for normally distributed data or median (P_25_, P_75_) for non-normally distributed data; BMI, body mass index. TNM, Tumour–Node–Metastasis.

### Comparison of CFS Scores Between Groups

Pre-nursing CFS scores did not differ significantly between the two groups (*p* = 0.985). Post-nursing CFS scores in the combined nursing group were significantly lower than those in the control group (*p* < 0.001, Table 3).

**Table 3.** **Comparison of CFS scores between groups**.

Variable	Time point	Control group (n = 100)	Combined nursing group (n = 100)	*t*	*p*
CFS	Pre-nursing	45.17 ± 7.18	45.15 ± 7.68	0.019	0.985
	Post-nursing	43.18 ± 7.28	35.27 ± 7.75****	7.439	<0.001

Note: Values are presented as mean ± SD; Compared with pre-nursing scores within the same group, ****p < 0.0001; CFS, Cancer Fatigue Scale.

### Comparison of CD-RISC Scores Between Groups

Pre-nursing CD-RISC scores did not differ significantly between the groups (*p* = 0.476). Post-nursing CD-RISC scores in the combined nursing group were significantly higher than those in the control group (*p* < 0.001, Table 4).

**Table 4.** **Comparison of CD-RISC scores between groups**.

Variable	Time point	Control group (n = 100)	Combined nursing group (n = 100)	*t*	*p*
CD-RISC	Pre-nursing	60.78 ± 7.04	60.07 ± 7.01	0.715	0.476
	Post-nursing	61.27 ± 7.69	72.60 ± 6.49****	–11.260	<0.001

Note: Values are presented as mean ± SD; Compared with pre-nursing scores within the same group, ****p < 0.0001; CD-RISC, Connor-Davidson Resilience Scale.

### Comparison of SAS Scores Between Groups

Pre-nursing SAS scores did not differ significantly between the two groups (*p* > 0.05). Post-nursing SAS scores in the combined nursing group were significantly higher than those in the control group (*p* < 0.001, Table 5).

**Table 5.** **Comparison of SAS scores between groups**.

Variable	Time point	Control group (n = 100)	Combined nursing group (n = 100)	Z	*p*
SAS	Pre-nursing	63.00	63.00	–0.507	0.612
		(61.00, 65.00)	(60.00, 64.25)		
	Post-nursing	62.00	55.00	–11.373	<0.001
		(61.00, 65.00)	(53.00, 57.00)****		

Values are presented as median (P_25_, P_75_); Compared with pre-nursing scores within the same group, ****p < 0.0001; SAS, Self-Rating Anxiety Scale.

### Comparison of FACT-G Scores Between Groups

Pre-nursing FACT-G scores were not significantly different between the two groups (*p* = 0.104). Post-nursing FACT-G scores were notably greater in the combined nursing group compared with controls (*p* < 0.001, Table 6).

**Table 6.** **Comparison of FACT-G scores between groups**.

Variable	Time point	Control group (n = 100)	Combined nursing group (n = 100)	*t*	*p*
FACT-G	Pre-nursing	63.09 ± 8.40	65.08 ± 8.80	–1.635	0.104
	Post-nursing	65.26 ± 7.76	73.01 ± 8.29****	–6.827	<0.001

Note: Values are presented as mean ± SD; Compared with pre-nursing scores within the same group, ****p < 0.0001; FACT-G, Functional Assessment of Cancer Therapy-General.

### Incidence of Adverse Reactions

The overall incidence of adverse reactions was 28.00% in the combined nursing group and 36.00% in the control group, with no statistically significant difference (*p* = 0.225, Table 7).

**Table 7.** **Comparison of adverse reaction rates between groups [n (%)]**.

Group	Fatigue	Drowsiness	Pain	Total
Control group (n = 100)	9(9.00)	5(5.00)	22(22.00)	36(36.00)
Combined nursing group (n = 100)	6(6.00)	7(7.00)	15(15.00)	28(28.00)
χ ^2^	0.649	0.355	1.625	1.471
*p*	0.421	0.552	0.202	0.225

Note: Values are presented as n (%).

### Correlation Analysis of Post-Nursing CFS, CD-RISC, SAS, and FACT-G

Spearman rank correlation analysis (Table 8) showed that post-nursing CFS scores were positively correlated with SAS scores (r = 0.407, *p* < 0.001) and negatively correlated with CD-RISC (r = –0.474, *p* < 0.001) and FACT-G scores (r = –0.330, *p* < 0.001). Post-nursing SAS scores were negatively correlated with CD-RISC (r = –0.560, *p* < 0.001) and FACT-G scores (r = –0.426, *p* < 0.001). Post-nursing CD-RISC scores were positively correlated with FACT-G scores (r = 0.298, *p* < 0.001).

**Table 8.** **Correlation analysis of post-nursing CFS, CD-RISC, SAS, and FACT-G**.

Variable	Post-nursing CFS	Post-nursing CD-RISC	Post-nursing SAS	Post-nursing FACT-G
Post-nursing CFS	1			
Post-nursing CD-RISC	–0.474 (<0.001)	1		
Post-nursing SAS	0.407 (<0.001)	–0.560 (<0.001)	1	
Post-nursing FACT-G	–0.330 (<0.001)	0.298 (<0.001)	–0.426 (<0.001)	1

Note: CFS, Cancer Fatigue Scale; CD-RISC, Connor-Davidson Resilience Scale; SAS, self-rating anxiety scale; FACT-G, Functional Assessment of Cancer Therapy-General.

### Path Analysis

**Fig. 1.**
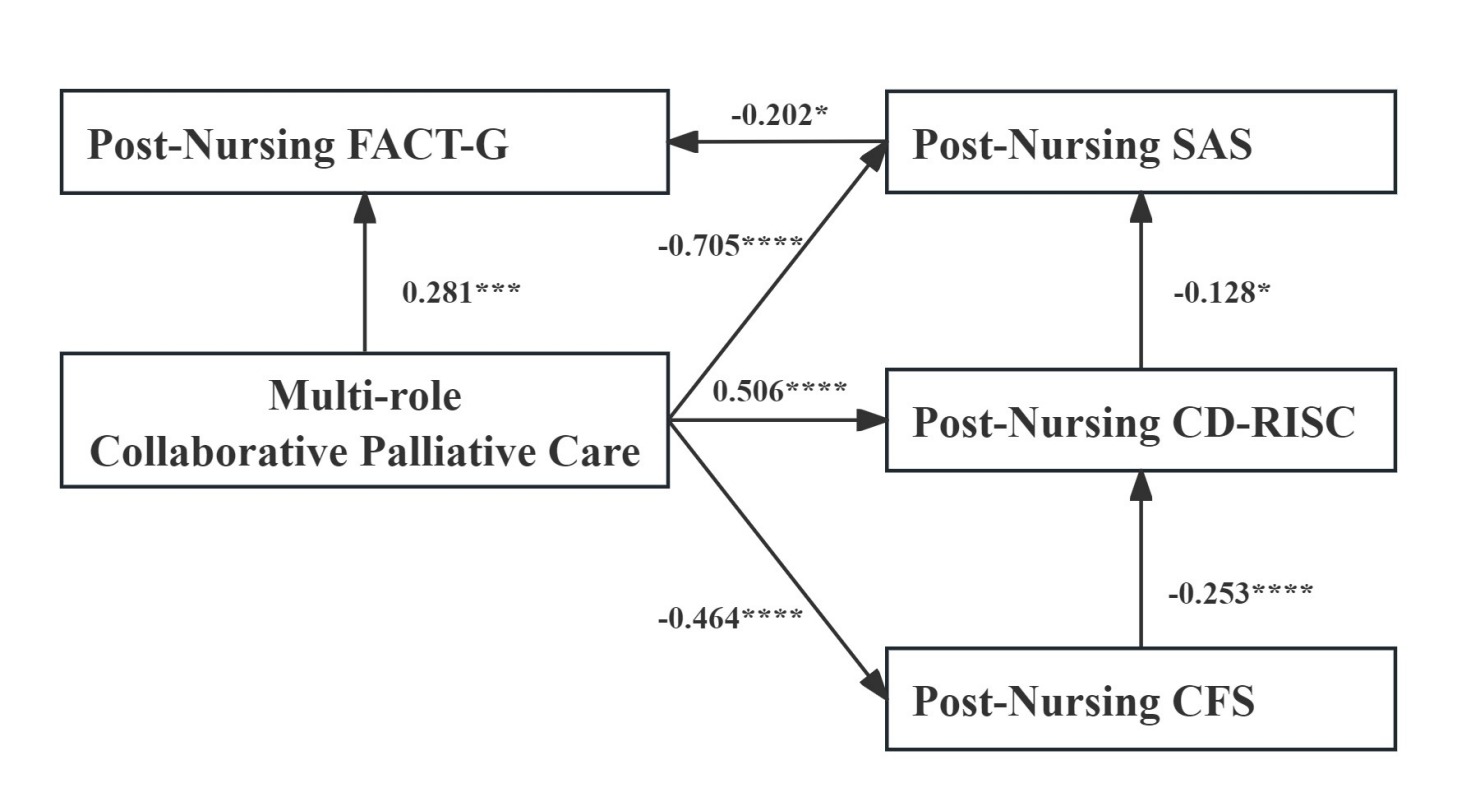
**Structural equation model**. FACT-G, Functional Assessment of Cancer Therapy-General; SAS, self-rating anxiety scale; CD-RISC, Connor-Davidson Resilience Scale; CFS, Cancer Fatigue Scale. *p < 0.05, ***p < 0.001, ****p < 0.0001.

Based on the results of nursing effectiveness verification and Spearman correlation analysis, multi-role collaborative palliative care, post-nursing SAS, CFS, CD-RISC, and FACT-G scores were included in the path analysis. The exploratory structural equation model was constructed to examine potential statistical associations. The model examined direct associations between multi-role collaborative palliative care and SAS, CFS, CD-RISC, and FACT-G. It also examined associations between CFS and CD-RISC, CD-RISC and SAS, and SAS and FACT-G, based on theoretical frameworks [21, 22]. AMOS software was used to analyse the proposed model structure. The results of the structural equation modelling path analysis were shown in Fig. 1 and Table 9. All main paths were significant, with *p* values < 0.05, confirming the proposed hypotheses. The structural equation model demonstrated good fit (χ^2^ = 4.705, *p* = 0.319, χ^2^/df = 1.176, GFI = 0.995, RMSEA = 0.030, CFI = 0.998, TLI = 0.995, NFI = 0.989, NNFI = 0.995, IFI = 0.998) (Table 10).

**Table 9.** **Summary of standardised path coefficients in the exploratory model**.

Independent variable	→	Dependent variable	Unstandardised β	SE	Z	*p*	Standardised β
Multi-role collaborative palliative care	→	Post-nursing FACT-G	4.958	1.786	2.776	0.006	0.281
Multi-role collaborative palliative care	→	Post-nursing SAS	–6.883	0.540	–12.735	0.000	–0.705
Multi-role collaborative palliative care	→	Post-nursing CFS	–7.910	1.068	–7.406	0.000	–0.464
Multi-role collaborative palliative care	→	Post-nursing CD-RISC	9.196	1.087	8.461	0.000	0.506
Post-nursing CFS		Post-nursing CD-RISC	–0.270	0.064	–4.232	0.000	–0.253
Post-nursing SAS	→	Post-nursing FACT-G	–0.364	0.183	–1.989	0.047	–0.202
Post-nursing CD-RISC	→	Post-nursing SAS	–0.069	0.030	–2.306	0.021	–0.128

Note: → indicates the direction of the modelled association; CFS, Cancer Fatigue Scale; CD-RISC, Connor-Davidson Resilience Scale; SAS, self-rating anxiety scale; FACT-G, Functional Assessment of Cancer Therapy-General.

**Table 10.** **Goodness-of-fit indices of the structural equation model**.

Common indicators	χ ^2^	df	p	χ^2^/df	GFI	RMSEA	TLI	CFI	NFI	NNFI	IFI
Judgement standard	-	-	>0.05	<3	>0.9	<0.10	>0.9	>0.9	>0.9	>0.9	>0.9
Value	4.705	4	0.319	1.176	0.995	0.030	0.995	0.998	0.989	0.995	0.998
Whether it meets the standards	yes	yes	yes	yes	yes	yes	yes	yes	yes	yes	yes

Note: df, Degrees of Freedom; GFI, Goodness of Fit Index; RMSEA, Root Mean Square Error of Approximation; TLI, Tucker-Lewis Index; CFI, Comparative Fit Index; NFI, Normed Fit Index; NNFI, Non-Normed Fit Index; IFI, Incremental Fit Index.

Path analysis showed that multi-role collaborative palliative care was positively associated with post-nursing FACT-G (β = 0.281, *p* < 0.01) and CD-RISC (β = 0.506, *p* < 0.001), and negatively associated with post-nursing SAS (β = –0.705, *p* < 0.001) and CFS (β = –0.464, *p* < 0.001). Post-nursing SAS showed a negative association with FACT-G (β = –0.202, *p* < 0.05). CFS was negative associated with CD-RISC (β = –0.253, *p* < 0.001), and CD-RISC was negative associated with SAS (β = –0.128, *p* < 0.05).

## Discussion

Radiation and chemotherapy are vital in treating advanced lung cancer. However, these therapeutic modalities are often prolonged, associated with significant adverse effects, such as myelosuppression, appetite loss, nausea and vomiting, and immunosuppression, and incur substantial financial costs. Such factors not only lead to substantial physiological harm but also impose remarkably psychological stress, triggering or exacerbating existing negative emotions and thereby adversely affecting treatment adherence and outcomes [23, 24]. Moreover, a study has shown that approximately 74% of patients with advanced lung cancer experience pain of varying degrees, with moderate to severe pain accounting for about 31% [25], which further exacerbates the physiological burden and induces CRF along with other adverse psychological outcomes, thereby severely impairing quality of life. These findings underscore the necessity of appropriate nursing measures to alleviate physiological symptoms and improve psychological well-being in this population.

The findings of this research demonstrates that multi-role collaborative palliative care can effectively reduce CRF, alleviate anxiety, and improve psychological status and quality of life in advanced lung cancer patients. These findings are consistent with the reports of Wang and Ding [26] and Yuan *et al*. [27]. This effect can be explained by the integrated care model, which centres on the palliative care principle of symptom relief and suffering alleviation, and delivers comprehensive nursing measures through multidisciplinary collaboration.

In terms of physiological aspects, physicians and nurses provide individualized antitumour and analgesic treatment according to patient condition, promptly addressing symptoms such as myelosuppression, anaemia, pain, and insomnia. Rehabilitation therapists implement respiratory training, moderate exercise, and functional exercises to enhance physical endurance and relieve dyspnoea. Dietitians optimise nutritional status to improve patients’ physical strength and immune function [28, 29, 30]. These nursing measures collectively mitigate physiological contributors to CRF, thereby reducing both the incidence and severity of fatigue [31].

Concerning psychological aspects, information sharing among physicians, nurses, and psychologists allows patients to be informed of their condition appropriately, with family consent, reducing information asymmetry that could trigger anxiety and fear. Nurses routinely monitor emotional status, provide encouragement, and promptly communicate abnormal findings to psychologists, facilitating precise psychological interventions and maximising improvements in psychological well-being while alleviating patients’ anxiety. Psychologists conduct daily counselling, organise patient support meetings and distraction-based activities, offering emotional comfort and social support to help patients maintain a positive mindset when facing illness. Furthermore, the care team delivers educational resources, guides family communication, and coordinates support, thereby diminishing psychological stress [32, 33].

Throughout the nursing measures, the multi-role collaborative palliative care model creates a mutually reinforcing network between physiological and psychological nursing measures. This integrated approach not only effectively reduces CRF and alleviates anxiety but also improves psychological resilience, ultimately enhancing overall quality of life in patients with advanced lung cancer. Although this study shows that multi-role collaborative palliative care has numerous benefits for patients, its application in clinical practice still faces certain challenges: this care model requires collaboration among multiple professional teams, but not all hospitals have the corresponding professionals, cross-professional communication and information sharing also present certain difficulties; moreover, this model has high nursing time, training, and resource requirements, and some hospitals with limited resources may find it difficult to fully implement it. To improve clinical feasibility, the team members can be flexibly configured according to the actual conditions of the hospital, standardised communication procedures and operation guidelines can be formulated, and a phased promotion strategy can be adopted, starting with pilot projects in better-equipped departments and then gradually expanding to other wards, in order to achieve the replicability and sustainability of the care model.

Path analysis indicated that among all paths, the standardised path coefficient for “multi-role collaborative palliative care → post-intervention SAS” was the largest in absolute value (β = —0.705), suggesting that this care model has the strongest statistical association with lower anxiety levels in patients with advanced lung cancer, with important academic significance. Moreover, the constructed model suggested that the effect of this care model on anxiety may not be limited to the direct effect, but could also operate through potential multiple indirect pathways, such as “relieving CRF → improving psychological status → reducing anxiety.” Possible mechanisms may be speculated as follows: first, through comprehensive interventions including emotional support, symptom management, nutritional guidance, and functional exercise, the care model can effectively alleviate patients’ physical discomfort and distress, reduce CRF, and thereby lessen the psychological burden caused by persistent fatigue, ultimately improving psychological status and indirectly reducing anxiety levels; second, the enhancement of psychological status can generally strengthen patients’ ability to cope with disease- and treatment-related stress, reducing anxiety in the face of disease uncertainty. As anxiety levels decrease, patients’ experiences in physiological, psychological, and social functioning improved, ultimately contributing to enhanced quality of life.

However, this study still has certain limitations. First, SEM analysis used cross-sectional data from a single post-intervention time point. Therefore, the directed paths represent statistical associations only and cannot establish temporal precedence or causal relationships. Future longitudinal studies with multiple time points are necessary to rigorously test potential mediating pathways. Second, this study is a single-centre retrospective study with a relatively small sample size. The research subjects were divided from previous clinical data, which to some extent may lead to selection bias, thereby affecting the representativeness and generalizability of the research results. Third, retrospective studies rely on historical medical records and scale assessment results. Some clinical information may have information bias due to inconsistent assessment time points or subjective differences, which may affect the measurement accuracy of related variables and the estimation of the relationships between variables in the model. Moreover, although there is no statistically significant difference in gender, age, and main clinical characteristics between the two groups, suggesting that the baseline data are comparable, there may still be unmeasured potential confounding factors, which may affect the relationships between variables. At the same time, under the current sample size conditions, including multiple covariates simultaneously may increase the complexity of the structural equation model, thereby affecting the stability of model fitting. In the future, multi-centre, large-scale prospective studies will be conducted, and while ensuring the stability of the model, age, gender, disease stage, and other known potential confounding factors, as well as other possible potential confounding factors, will be included in the structural equation model to further verify the robustness and universality of the variable relationships and action paths revealed in this study.

## Conclusions

In summary, the application of multi-role collaborative palliative care in patients with advanced lung cancer helps alleviate CRF, reduce anxiety, improve psychological well-being, and enhance quality of life, demonstrating significant clinical applicability. Furthermore, based on data from patients receiving this comprehensive nursing model, exploratory path analysis indicates that multi-role collaborative palliative care, with anxiety relief as a core outcome, shows significant statistical associations with lower CRF and better psychological status, which are in turn associated with lower anxiety. These findings provide important insights into the potential mechanisms of nursing interventions and offer valuable guidance for optimising clinical care strategies.

## Availability of Data and Materials

The datasets used and analyzed during the current study are available from the corresponding author on reasonable request.
